# Compliance of the fish outflow tract is altered by thermal acclimation through connective tissue remodelling

**DOI:** 10.1098/rsif.2021.0492

**Published:** 2021-11-17

**Authors:** Adam N. Keen, John J. Mackrill, Peter Gardner, Holly A. Shiels

**Affiliations:** ^1^ Faculty of Biology, Medicine and Health, University of Manchester, Manchester, UK; ^2^ Cardiovascular Medicine, Radcliffe Department of Medicine, University of Oxford, Oxford, UK; ^3^ Department of Physiology, University College Cork, Cork, County Cork, Ireland; ^4^ School of Chemical Engineering and Analytical Science, Manchester Institute of Biotechnology, University of Manchester, UK

**Keywords:** bulbus arteriosus, stiffness, collagen, elastin, matrix metalloproteinase, Fourier transform infrared spectroscopy

## Abstract

To protect the gill capillaries from high systolic pulse pressure, the fish heart contains a compliant non-contractile chamber called the bulbus arteriosus which is part of the outflow tract (OFT) which extends from the ventricle to the ventral aorta. Thermal acclimation alters the form and function of the fish atria and ventricle to ensure appropriate cardiac output at different temperatures, but its impact on the OFT is unknown. Here we used *ex vivo* pressure–volume curves to demonstrate remodelling of passive stiffness in the rainbow trout (*Oncorhynchus mykiss*) bulbus arteriosus following more than eight weeks of thermal acclimation to 5, 10 and 18°C. We then combined novel, non-biased Fourier transform infrared spectroscopy with classic histological staining to show that changes in compliance were achieved by changes in tissue collagen-to-elastin ratio. *In situ* gelatin zymography and SDS-PAGE zymography revealed that collagen remodelling was underpinned, at least in part, by changes in activity and abundance of collagen degrading matrix metalloproteinases. Collectively, we provide the first indication of bulbus arteriosus thermal remodelling in a fish and suggest this remodelling ensures optimal blood flow and blood pressure in the OFT during temperature change.

## 1. Introduction

The fish circulatory system is a single circuit. Upon leaving the heart, blood passes through an outflow tract (OFT) leading to the ventral aorta which takes blood to the gill where it is oxygenated before continuing around the body perfusing the tissues before returning to the heart [1]. Despite close proximity to the gill, strong ventricular contraction is required to eject blood at adequate pressure to circulate the body. Therefore, to prevent damage of the delicate gill capillary network, the OFT of teleost fish is highly specialized and is composed of a chamber called the bulbus arteriosus and bulbo-ventricular valves [2]. The bulbus arteriosus acts like a windkessel absorbing the energy of systolic blood through elastic expansion and recoil smoothing and steadying pulsatile blood flow across gill capillary networks and throughout the body [3–5]. Although the terms ‘OFT’ and ‘bulbus arteriosus’ are often used interchangeably, for clarity here we define the bulbus arteriosus as the chamber and the OFT as the whole outflow section between the ventricle and the ventral aorta.

The bulbus arteriosus is considered an evolutionary novelty in the teleost lineage with anatomical and histological properties that are very different from those found in the OFTs of other vertebrates including elasmobranchs. Although considered part of the heart, the teleost OFT does not contain striated muscle rather it is structurally similar to an artery [5–7]. Its endocardial layer consists of bulbular ridges lined with endothelial cells, and the inner media is an elastic matrix with inter-dispersed smooth muscle cells. However, unlike an artery, the elastin fibres are not organized into lamellae but are arranged as fibrils [5]. The fibrillar arrangement and high elastin-to-collagen ratio make the bulbus arteriosus very compliant, meaning it has an ‘r-shaped’ static inflation curve compared to the typical ‘j-shaped’ inflation of an artery [2,3,8–18]. The outer media integrates sub-epicardial layers of collagen fibres surrounded by mesothelial cells, which serve to prevent damage from high-pressure blood flow. The specialized elastic matrix of the bulbus arteriosus make it both strong and compliant which allows it to withstand high haemodynamic strain while limiting the pulsatile nature of blood flow [7].

Rainbow trout experience seasonal fluctuations in ambient temperature but remain active throughout the year. Low-temperature slows heart rate, decreases contractile function and increases blood viscosity [19,20]. These temperature-induced changes in pressure and volume load cause dynamic remodelling of the fish ventricle [21–23] and atrium [24] but corresponding changes in the OFT have not been investigated. Cold acclimation increases ventricular mass to facilitate the pumping of higher volumes of viscous blood. This ventricular hypertrophy has been linked to elevated central venous pressure, end-diastolic volume and increased stroke volume ejected into the OFT [25–28]. To assess whether the increased cardiac load translates to functional differences in the passive properties of the fish OFT we investigated the effects of cold acclimation (from 10 ± 1°C to 5 ± 1°C) and warm acclimation (from 10 ± 1°C to 18 ± 1°C) on the compliance and connective tissue structure of the rainbow trout OFT. We hypothesized remodelling would increase stiffness in the cold and increase compliance in the warm and that these differences would be mediated via changes in collagen and elastin. We used zymography to understand regulation of collagen and novel and non-biased Fourier transform infrared (FTIR) spectroscopy in conjunction with classic histological staining to test the hypothesis that collagen order and coherency were impacted by thermal acclimation and that changes in these structural elements underlie functional changes in the chamber.

## 2. Material and methods

### 2.1. Experimental animals

Sexually mature female rainbow trout (*Onchorynchus mykiss; n* = 48; table 1) were housed on a 12 h light : 12 h dark cycle in approximately 500 l re-circulated aerated, de-chlorinated freshwater tanks at 10 ± 1°C and fed to satiation three times per week. Fish were held under these conditions for a minimum of two weeks before being randomly assigned to one of three acclimation groups; cold (5 ± 1°C), control (i.e. no change; 10 ± 1°C) or warm (18 ± 1°C). Water temperature of the warm and cold acclimation groups was changed by 1°C per day until desired temperature was reached and then held for a minimum of eight weeks before experimentation. The photoperiod for the cold-acclimated animals was changed to 8 h light : 16 h dark cycle to simulate winter [19].

**Table 1.** Heart chamber and body mass morphometrics. *Note*: RHM, relative heart mass; OFT, outflow tract; ROFTM, relative outflow tract mass. *N* = 48, 16 female fish per acclimation group.

	cold-acclimated (5°C)	control (10°C)	warm-acclimated (18°C)
body mass (g)	480 ± 64.3	526 ± 42.8	524 ± 60.2
heart mass (g)	0.94 ± 0.09	1.15 ± 0.12	0.99 ± 0.11
RHM (g body mass^−1^ × 100)	0.21 ± 0.01	0.22 ± 0.01	0.19 ± 0.01
OFT mass (g)	0.15 ± 0.02	0.16 ± 0.02	0.12 ± 0.01
ROFTM (g body mass^−1^ × 100)	0.03 ± 0.0002	0.03 ± 0.0001	0.03 ± 0.0002

Fish were killed by a blow to the head followed by severance of the spinal cord and destruction of the brain. The heart was excised and used either for *ex vivo* pressure volume curves or processed for further analysis as described below. All holding conditions and experimental procedures were approved by the Ethical Review Board of the University of Manchester.

### 2.2. *Ex vivo* passive pressure–volume curves

Passive (i.e. static) pressure–volume curves were generated following the methodology of [21,24]. Briefly, the intact isolated heart was placed into an organ bath containing a physiological saline solution (150 mM NaCl, 5.4 mM KCl, 2 mM CaCl_2_, 1.5 mM MgSO_4_, 0.4 mM NaH_2_PO_4_, 10 mM HEPES, 10 mM glucose at pH 7.7) at 10 ± 1°C to which 20 mM 2,3-butanedione monoxime (BDM) was added to prevent cross-bridge cycling. Pressure–volume curves from OFTs of rainbow trout from each acclimation group were generated at a common temperature, of 10 ± 1°C, to isolate the chronic remodelling effects from the acute effects of temperature on passive properties. A cannula was fed through the ventricle into the lumen of the OFT and secured at the bulbus–ventricular junction using 0–0 silk thread (Harvard Apparatus, Holliston, MA, USA). An atraumatic clamp was placed at the arterial end of the bulbus making the OFT an ‘empty’ sealed chamber with the cannula inside and pressure set to zero. The cannula was connected to a syringe pump (INFORS AG, Bottmingen, CHE), in series with a pressure transducer, containing 10 ± 1°C physiological saline solution with BDM and a small amount of blue food colouring. The pressure transducer was calibrated daily against a static water column and recorded at 1000 Hz (Chart5, PowerLab, ADI Instruments, Dunedin, New Zealand). The physiological saline solution was pumped into the OFT at 0.05 ml min^−1^ until maximum volume was achieved, determined by visual leak of the saline containing blue dye and a drop in the pressure trace.

### 2.3. Tissue processing

Excised OFTs were bisected with one half snap frozen in OCT and stored at −80°C. The other half was fixed in 10% neutral buffered formalin solution (Sigma-Aldrich, St Louis, MO, USA) and embedded in paraffin wax so that sections could be cut in the transverse/axial plane. As the thickness of the outer media and morphology of the inner media are not consistent throughout the length of the bulbus, we were careful to section only the middle portion of each bulbus for histological comparison between temperature groups [18].

### 2.4. Fourier transform infrared spectroscopy

Fixed OFT tissue from warm- and cold-acclimated animals were analysed using transmission mode FTIR imaging spectroscopy using a Varian 670-IR spectrometer coupled with a Varian 620-IR imaging microscope (Agilent Technologies, CA, USA). Our principal aim with these analyses was to understand if collagen became more or less ordered with thermal acclimation which could be detected by subtle changes in spectra. We did not perform FTIR analysis on control tissue due to equipment constraints and because the expectation was that spectral differences would be small and would be greatest between the warm and cold acclimated groups. Data from warm- and cold-acclimated animals were collected in the 950–3800 cm^−1^ range, at a spectral resolution of 5 cm^−1^, with the co-addition of 96 scans for sample spectra and 256 scans for the background spectra. For each tissue section, spectra were collected at 50 areas across the tissue.

Infrared spectral data were imported into MATLAB (MATLAB 2014a, Mathworks, USA) and quality tested by the amide I region (1597–1738 cm^−1^). The absorbance values were used to determine which spectra were accepted or rejected and were determined separately for each hyperspectral image by reference to an image of the tissue section. The band associated with ambient gas-phase CO_2_ was removed, as was any data outside of the specified wavenumber range (950–1750 cm^−1^). The reason for restricting the analysis to this range is that it is spectrally much richer than the high wavenumber region and is where the main vibrational bands of collagen lie. Data were subjected to principle component analysis (PCA) noise reduction [29,30], vector normalization (to account for variations in the thickness of the tissue, and RMieS-EMSC correction (to account for the effects of Mie scattering) using 100 iterations of the algorithm and a Matrigel^TM^ spectrum as the initial reference point [31]. The 50 individual spectra for each tissue were averaged to provide mean spectra for each tissue section. Following analysis of mean spectra, data were transformed into the second derivative.

### 2.5. Connective tissue histology

Fibrillar collagen and elastin content were analysed semi-quantitatively following the methodology of [21,24]. Briefly, formalin-fixed and paraffin-embedded tissue samples were sectioned at 5 µm (Leica CM3050S cryostat, Leica, Wetzlar, Germany) and mounted onto glass slides (Super frost plus, Thermo Fisher Scientific, Waltham, MA, USA). Serial sections from each sample were stained with picro-sirus red for collagen and Miller's elastic stain for elastin. Picro-sirus red images were quantified using polarized light microscopy and Miller's elastic images were quantified using bright-field microscopy (Leica, Wetzlar, Germany) as previously described [21,24]. Mean fibrillar collagen and elastin contents were expressed as a percentage of total tissue cross-sectional area, determined using ImageJ [32]. Quantitative analyses of collagen orientation (coherency) were conducted on picro-sirus red stained tissue using ImageJ with the OrientationJ plug-in, following previously published methodology [33,34]. Three tissue sections were considered for each individual. Collagen and elastin was quantified as a % of total tissue in each section. On each tissue section, three separate image montages were taken across the full diameter of the cross section. All histological analysis was conducted blind to the acclimation group.

### 2.6. Matrix metalloproteinase gelatin zymography

To characterize the *abundance* and activation of specific matrix metalloproteinases (MMPs), we used SDS-PAGE-based gelatin zymography. Snap frozen OFT from four fish for each acclimation group were rinsed with PBS, then protein was extracted in 10 volumes per wet weight of 0.05% Brij-35, 10 mM CaCl_2_, 50 mM Tris–HCl pH 7.4 on ice, using three 10 s bursts of an MSE Soniprep150 sonicator (exponential probe, 10 µm amplitude). Extracts were cleared by centrifugation at 10 000*g* for 10 min and protein content was determined using the Bradford assay with bovine serum albumin as a standard. Equal quantities of protein (1 µg per lane) from each fish for each condition were analysed by gelatin SDS-PAGE, as described by Lødemel *et al*. [35] to allow the use of band intensity as an estimate of protein abundance. Conditioned media from HepG2 cell cultures (100 ng protein per lane) which is a hepatocyte-like cell line that secretes predominantly pro-MMP-2 [36] and recombinant active human MMP-2 (1 ng protein per lane, Millipore) were used as positive controls and the assignment of each band on the gel was based on their apparent molecular weight and published data on other fish [35]. The abundance of each gelatinase band was measured using the ‘Gel’ function of ImageJ.

### 2.7. *In situ* matrix metalloproteinase gelatin zymography

The *activity* of endogenous MMP gelatinases were semi-quantitatively analysed by *in situ* zymography of tissue cryosections, following the methodology of [37]. Frozen tissue was sectioned at 10 µm and mounted onto glass slides. DQ gelatin (porcine; Invitrogen, Thermo Fisher Scientific, Waltham, MA, USA) was dissolved in dH_2_O (to a concentration of 1 mg ml^−1^) and diluted 1 : 10 in low-temperature gelling agarose solution (Sigma-Aldrich, St Louis, MO, USA; 10 mg ml^−1^ in PBS) with DAPI (1 µg ml^−1^) at 37°C. Forty microlitres of agarose/DQ gelatin was added to each tissue section and a coverslip placed on the slide to ensure even film thickness across the sample section. Samples were imaged using a FITC filter (Leica, Wetzlar, Germany). To account for tissue auto-fluorescence, negative control slides were used to determine the microscope settings for each section and all imaging was done in a single sitting. Three tissue sections were considered for each individual OFT. On each tissue section, three separate image montages of the same area were taken across the full diameter of the cross section. Following background subtraction, mean fluorescence intensity was calculated for each image, analysed using ImageJ. All analysis was conducted blind to the acclimation group.

### 2.8. Statistical analyses

Chamber filling volume was calculated from the filling time as described in [21,24]. The effect of temperature acclimation on the pressure–volume relationship of the OFT was assessed by ANCOVA with pressure as the dependent variable, volume and acclimation group as fixed factors and body mass as the covariate, with a Tukey post-hoc test for differences between groups, using R. The model was performed on data below 10 kPa, which approximates the maximum physiological pressures experienced by this species [38,39]. Chamber compliance (the change in volume for a given change in pressure standardized to body mass; ml kg^−1^ kPa^−1^) was calculated as the slope of the pressure–volume curve within the normal physiological range of this species [39]. Chamber distensibility (fold change in compliance) was calculated as the chamber compliance normalized to the chamber volume at onset of the physiological pressure range (i.e. 2 kPa) [38]. Differences in chamber compliance and distensibility, and collagen and elastin tissue content were assessed by GLM with Holm–Sidak post-hoc test for differences between groups using Prism v6 (GraphPad, San Jose, CA, USA). For all analyses significance was set at *p* < 0.05. Values are presented as mean ± s.e.m. throughout unless otherwise stated. Statistical details are provided in the figure legends.

## 3. Results

### 3.1. Thermal remodelling of *ex vivo* chamber compliance

**Figure 1.**
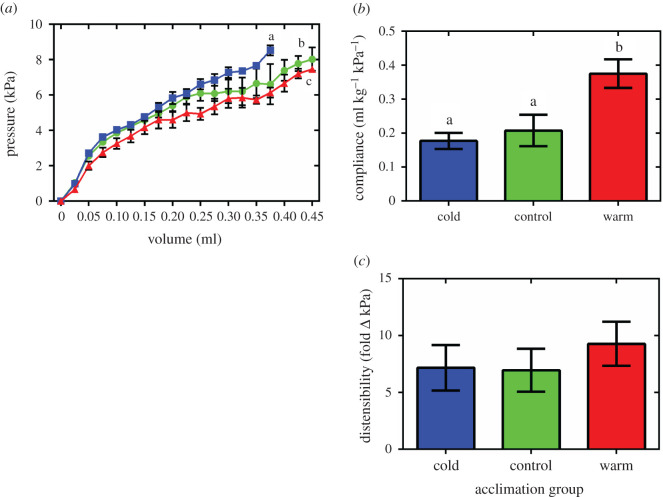
Compliance and distensibility of the fish OFT. (*a*) Mean passive pressure–volume relationships. The curves are shown within the physiological pressure range for this chamber (less than 10 kPa) and represent mean data. Pressure has been standardized to start at 0 kPa for graphical representation. (*b*) Maximum compliance (the change in volume for a given change in pressure) and (*c*) maximum distensibility (fold change in compliance) generated from the OFT for cold- (5°C; blue), control- (10°C; green) and warm- (18°C; red) acclimated rainbow trout (*n* = 8). Values are mean ± s.e.m. Significant differences in pressure–volume relationships were assessed by ANCOVA with volume as the dependent variable, acclimation group and pressure as the fixed factors and chamber mass as the covariate. Significant differences in chamber compliance and distensibility were assessed by ANOVA with a Holm–Sidak post-hoc test. Significance between groups is indicated by dissimilar letters (for (*a*) *p* < 0.0001 and for (*b*) *p* = 0.0082).

We generated *ex vivo* passive filling curves from freshly isolated intact OFT from fish from each acclimation group at a common test temperature of 10°C. Consistent with previous studies [5,38,39], the mean pressure–volume trace from each group in our study showed an ‘r-shaped’ profile (figure 1*a*). However, thermal acclimation altered the pressure–volume relationship during passive filling of the OFT (*R*^2^ = 0.68, *F*_2,8971_ = 160.95, *p* < 0.0001) showing increased stiffness following cold acclimation and increased compliance after warm acclimation compared to the controls (*t*-ratio = 26.81; figure 1*a*). We calculated chamber compliance within by the profile of the curve that corresponds to the *in vivo* physiological pressure range of the chamber (2–5 kPa) and found that it was increased by approximately 1.9-fold following warm acclimation compared to the control and cold-acclimated fish (figure 1*b*; *p* = 0.0082). However, we found no effect on chamber distensibility with thermal acclimation (figure 1*c*). At pressures nearing the upper limit of the chamber (approx. 10 kPa), filling profiles of the OFT have been shown to exhibit a steep increase in pressure [5,38]. In cold-acclimated fish, this characteristic pressure increase appeared to begin at a lower filling volume and, conversely, at a higher filling volume for warm-acclimated fish (figure 1*a*), however, this was not resolvable statistically. Taken together these results show that fish alter OFT compliance in response to thermal acclimation.

### 3.2. Thermally dependent changes in biochemical landscape

**Figure 2.**
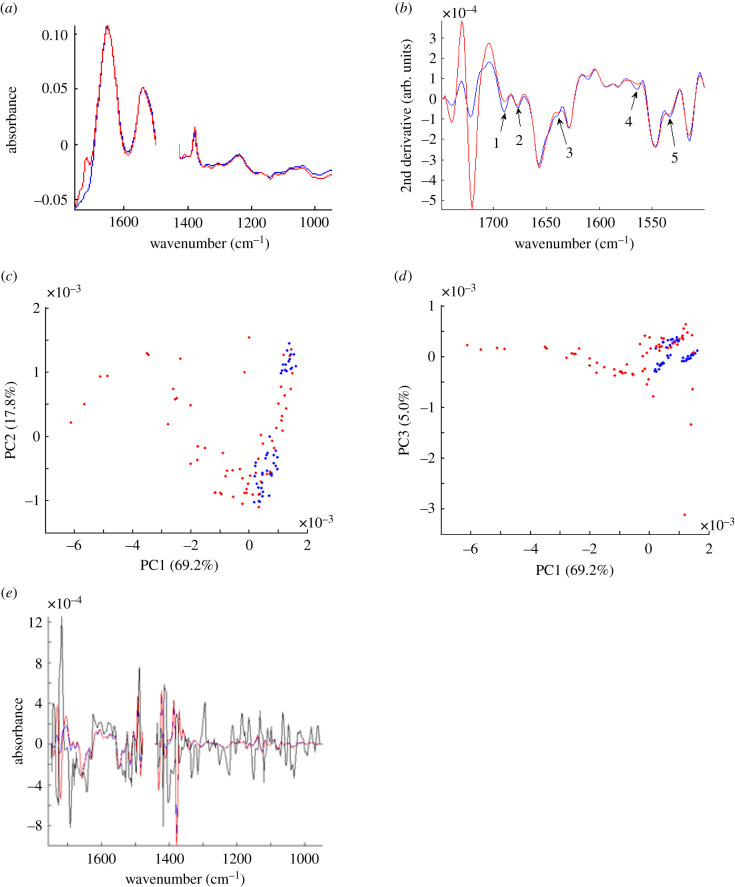
Infrared spectra and PCA analysis of fixed OFT tissue. (*a*) Mean Fourier transform infrared spectral data for cold- (blue) and warm-acclimated (red) OFT (*n* = 6, with 50 scan areas across each tissue section). (*b*) Second derivative mean spectral data showing deconvolution of amide peaks. Arrows indicate absorption peaks at wavenumbers of interest for ECM components (see text for details), 1 = 1690, 2 = 1675, 3 = 1640, 4 = 1565, 5 = 1535. Principal component analysis (PCA) of the second derivative, *n*th root spectra for cold- and warm-acclimated OFT showing separation along (*c*) PC1 against PC2 and (*d*) PC1 against PC3. (*e*) The second derivative of mean spectra with PC loadings plot (black).

Long-term temperature acclimation is associated with a change in the biochemical profile of heart tissue resulting from changes in protein composition, metabolic status and metabolite storage of the tissue [40] which can be assessed using non-biased FTIR spectroscopy. Indeed, FTIR has recently been used to characterize pathological changes in collagen fibre density associated with disease [41–43]. We investigated the mean spectral profiles from cold- and warm-acclimated fish bulbus arteriosus which showed the characteristic vibrational absorptions of collagen I (1080, 1377, 1429, 1611, 1628, 1657, 1678, 1690 cm^−1^) (figure 2*a*). In addition, we noted increased absorption at 1719 cm^−1^ in the warm-acclimated tissue that was not present in the cold-acclimated tissue (figure 2*a*). Minor acclimation-dependent differences in the amide III region (1330–1180 cm^−1^) were also observed with increased absorption in the warm-acclimated tissue from 1380 to 1340 and a peak at 1410 cm^−1^, but a higher absorption in the cold-acclimated tissue from 1340 to 900 cm^−1^ (figure 2*a*).

To further delineate thermally dependent alteration the spectral regions corresponding to collagen [44,45] we applied a second derivative to the mean spectra of the amide I and II bands (1700–1500 cm^−1^) [46] (figure 2*b*). There were changes in the amide I band, with greater absorption of the cold-acclimated tissue at 1690 and 1675 cm^−1^ and a change in shoulder shape at 1640 cm^−1^, which correspond to the vibrational modes for α-helices and β-turn structures, respectively [47,48]. There were also some changes at the amide II band, with greater absorption in the cold-acclimated tissue at 1565 cm^−1^ and a change in shoulder shape at 1535 cm^−1^. Interestingly, there is also a complete change in spectrum shape of the amide III band (from 1395 to 1415 cm^−1^) with a reduced absorption in the cold, a peak shift and a loss of an absorption shoulder.

The PCA result for the second derivative of the bulbus arteriosus spectra showed separation of the cold-acclimated fish by PC1 (69.2%) against PC2 (17.8%) and PC3 (5.0%) (figure 2*c,d*). The overall effect of the PC clustering of the data can be interpreted based on the profile of their PC loadings (figure 2*e*). PC loadings are correlation coefficients between the PC scores and their original variables. They measure the importance of each variable accounting for variability. When the loadings for PC1 were plotted on the same graph as the second derivative of the bulbus arteriosus spectra (figure 2*e*), there was strong variance at CH_3_ bending modes (1402 and 1404 cm^−1^), C–H deformation (1418 cm^−1^), the amide I band (1643 and 1692 cm^−1^) and the fatty acid ester (1726 cm^−1^) [49]. Together, these results suggest a change in the overall biochemical landscape between the cold- and warm-acclimated bulbus arteriosus with spectral differences in areas corresponding to extracellular matrix (ECM) protein vibrational signatures. The strong band at 1726 cm^−1^ in the warm-acclimated fish is indicative of an increase in fatty acid which was not expected.

### 3.3. Connective tissue content and fibre orientation coherency

**Figure 3.**
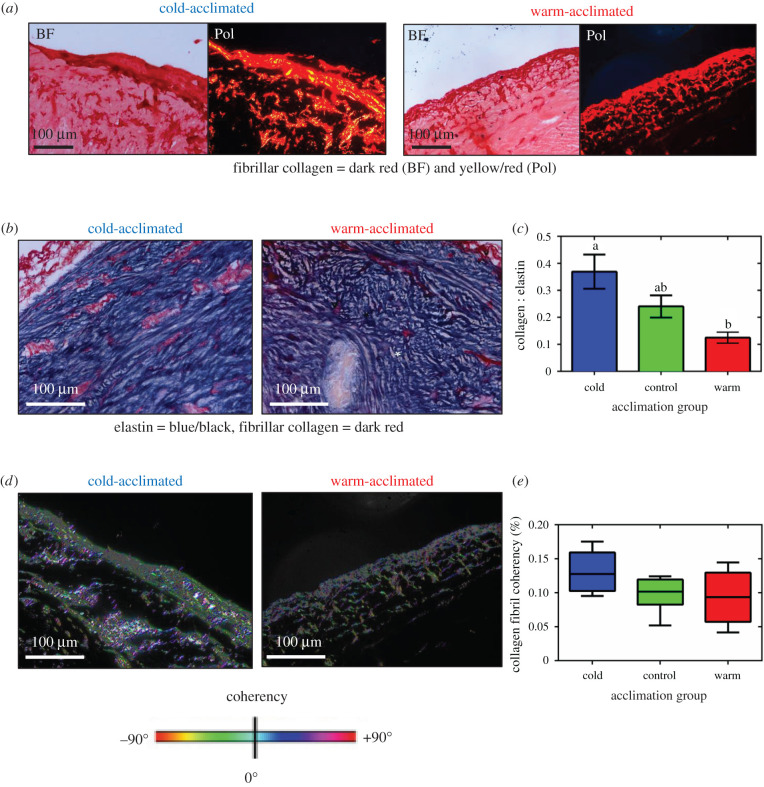
Connective tissue and fibrillar collagen orientation in the bulbus arteriosus. (*a*) Representative bright-field (BF) and plane-polarized light (Pol) images of cold- and warm-acclimated fish bulbus arteriosus, stained with picro-sirus red which labels collagen fibres dark red in BF and yellow/red under polarized light. (*b*) Representative BF images of cold- and warm-acclimated fish bulbus arteriosus stained with Miller's elastic stain. (*c*) Semi-quantitative analysis of the collagen-to-elastin ratio of the fish OFT. (*d*) Representative polarized light images for cold- and warm-acclimated bulbus arteriosus tissue sections, stained with picro-sirus red and assessed using OrientationJ. Fibre orientation is shown by colour, with fibres of the same colour showing coherency (see key below images). (*e*) Quantification of coherency of organized fibrillar collagen. Values are in (*c*) mean ± s.e.m. and in (*e*) as mean ± interquartile range with *n* = 8 fish for each acclimation temperature. Significant differences in the collagen-to-elastin ratio and collagen fibre coherency were assessed by ANOVA analysis with a Holm–Sidak post-hoc test. Significance between groups is indicated by dissimilar letters (*p* = 0.0086).

Cardiac remodelling due to changes in pressure or volume is often associated with alterations in cardiac ECM [21,24,50,51]. Previous studies have shown integration of collagen fibres in the media or luminal layers is minimal, but prevalent in the outer media and the outer adventitia [2,5,8,9]. We found that with cold acclimation there was an increase in dark red fibres in the inner layers of the bulbus arteriosus stained with picro-sirus red (figure 3*a*). Picro-sirus red has birefringent properties when imaged under plane-polarized light with collagen fibres appearing yellow/red. Cold-acclimated bulbus arteriosus showed increased birefringence (16.2 ± 3.1% collagen) compared to warm-acclimated (9.3 ± 5.7% collagen) and control (9.6 ± 5.0% collagen) bulbus arteriosus, suggesting an increased density of fibrillar collagen. While density of collagen fibres regulates chamber stiffness, elastin fibres mediate chamber compliance. We stained bulbus arteriosus sections with Miller's elastic stain, which labels elastin fibres dark-blue/black and collagen fibres dark red [52]. In accordance with our picro-sirus red staining, the cold-acclimated fish showed increased collagen fibre density compared to warm-acclimated fish (figure 3*b*). In addition, there was an increase in the intensity of blue–black staining in warm-acclimated fish (66.5 ± 6.6% elastin) suggesting increased elastin compared with the control (45.1 ± 9.9% elastin) and warm-acclimated fish (44.9 ± 7.2% elastin). By combining the results for collagen and elastin fibre quantification, we found that thermal acclimation led to changes in connective tissue content with a collagen-to-elastin ratio that was 1.4-fold higher in cold- and a 2.3-fold lower in warm-acclimated animals, compared with controls (figure 3*c*; *p* = 0.0086).

In addition to collagen fibre density, the organization of collagen fibres can affect overall tissue stiffness [33]. To investigate if thermal acclimation altered collagen fibre alignment in the fish bulbus arteriosus, we stained tissue sections with picro-sirus red, imaged under polarized light and measured fibre coherency using OrientationJ (figure 3*d*). There was no significant effect of temperature on coherency (*p* = 0.1; figure 3*e*). Taken together, these results show that the altered compliance of the bulbus arteriosus in response to thermal acclimation is due, at least in part, to changes in the composition of the ECM.

### 3.4. Characterization and endogenous gelatinase activity of matrix metalloproteinases

**Figure 4.**
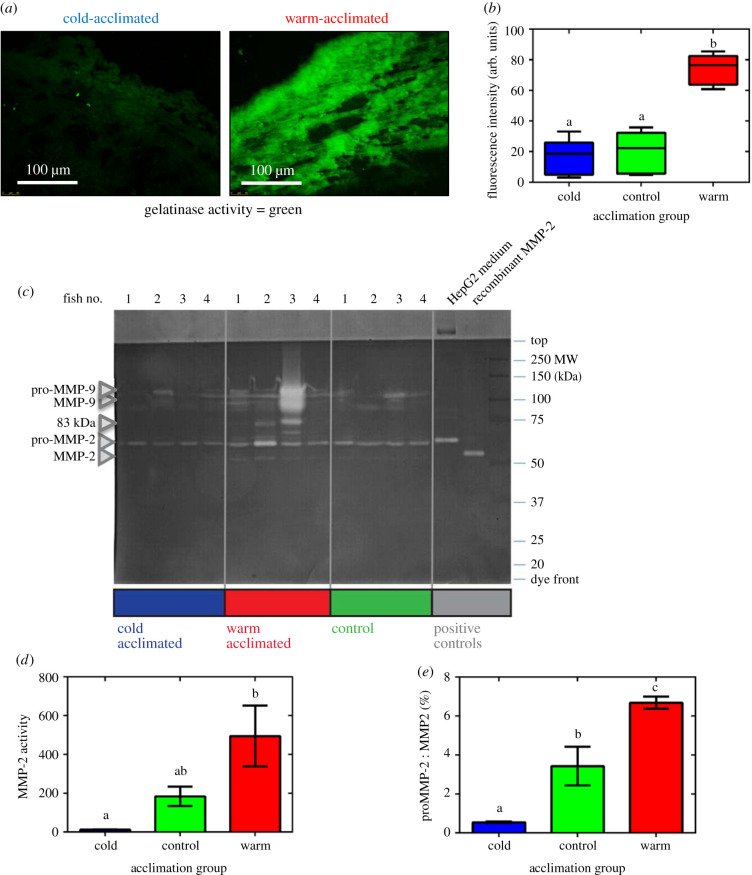
Characterization of endogenous matrix metalloproteinase (MMP) activity. (*a*) Representative fluorescent images to show *in situ* endogenous MMP gelatinase activity (green) in cold- and warm-acclimated fish bulbus arteriosus. (*b*) Semi-quantitative gelatinase zymography. Values are presented as mean ± interquartile range and range with *n* = 8 fish for each acclimation temperature. (*c*) Representative Coomassie R250 stained zymogram, indicating the relative molecular weights and abundances of gelatinases in OFT extracts from cold (5°C; blue), warm (18°C; red) and control (10°C; green) acclimated rainbow trout. Each lane is a separate fish for each of the three acclimation groups. The positions of pro-MMP-2 and MMP-2 and an uncharacterized 83 kDa gelatinase, are indicated by arrows on the left-hand side of the gel. HepG2 medium (from a cell line that secretes predominantly pro-MMP-2) and recombinant active human MMP-2 served as positive controls (see Material and methods for details). (*d*) Quantification of abundance of MMP-2 and (*e*) the ratio of proMMP-2 to MMP-2 abundance. Values are mean ± s.e.m. with *n* = 8 fish for each acclimation temperature. Significant differences were assessed by ANOVA and ANOVA on ranks with a Holm–Sidak post-hoc test. Significance between groups is indicated by dissimilar letters (for (*b*) *p* < 0.0001, for (*d*) *p* = 0.017, for (*e*) *p* = 0.0002).

ECM collagen content is a balance of deposition and degradation, with degradation regulated by MMPs [53,54]. We investigated the effect of endogenous total MMP gelatinase activity by *in situ* zymography of bulbus arteriosus tissue cryosections. This technique uses DQ gelatin to label the spatially localized gelatinase activity of total endogenous MMPs in tissue. We found an overall increase in green fluorescence, in warm- compared with cold-acclimated tissue, that was particularly evident in the collagen-rich outer layers of the bulbus arteriosus (figure 4*a*). Accordingly, semi-quantitative analysis of the fluorescence revealed a greater than fourfold increase in MMP activity in the warm-acclimated fish, compared to controls and cold-acclimated fish (figure 4*b*, *p* < 0.0001).

MMPs are initially synthesized as inactive pro-forms, the pro-domain must be removed to activate the enzyme [55]. To assess the contribution of specific MMPs, we used gelatin SDS-PAGE zymography. We found the presence of MMPs with apparent molecular weights similar to those of human proMMP-9, activated MMP-9 and proMMP-2 and activated MMP-2 in trout OFT tissue (figure 4*c*). In addition, an 83 kDa gelatinase that did not vary significantly with thermal acclimation was detected as has been previously described in the heart Atlantic cod (*Gadus morhua*) [35]. In some lanes, 75–60 kDa bands are also evident which may be new or modified gelatinases/collagenases however their molecular identity is currently unknown. Activated MMP-2 abundance was 39.3-fold higher than controls following warm acclimation (figure 4*d*, *p* = 0.017). Accordingly, the ratio of proMMP-2 to MMP-2, and hence level of MMP-2 activation, was twofold higher in warm-acclimated tissue than controls and 6.2-fold lower in cold-acclimated tissue than controls (figure 4*e*, *p* = 0.0002). There was a trend towards an increase in MMP-9 in the warm-acclimated fish, but the variation between individual fish meant that this did not reach statistical significance. Taken together, these results suggest that thermal remodelling of OFT ECM is controlled by increasing MMP-2 activity and abundance in response to warm temperatures and reducing MMP-2 activity and abundance in response to cold temperatures.

## 4. Discussion

For ectothermic species acute and chronic temperature changes present a significant challenge to the heart as they affect whole-body metabolism as well as cardiac contractility and can alter cardiac load via changes in blood viscosity [56]. Therefore, many fish that inhabit temperate waters undergo a seasonal, compensatory cardiac remodelling response to maintain appropriate heart function across temperatures. Here, we assessed the effect of long-term (greater than eight weeks) temperature acclimation on the rainbow trout bulbus arteriosus and OFT. Chronic cold caused OFT stiffening due to an increase in the collagen-to-elastin ratio of the bulbus arteriosus in comparison with chronic warming which caused increased compliance and a decrease in the collagen-to-elastin ratio. Changes in collagen content were associated with subtle changes in FTIR spectra and gelatinase MMP activity following thermal acclimation. Our results show that the passive and elastic nature of the bulbus arteriosus is regulated during thermal acclimation via structural and functional remodelling. Smoothing the pulsatile high-pressure systolic blood flow as stroke volume changes across temperatures and seasons may be driving remodelling of the bulbus arteriosus. In turn, increases in bulbus arteriosus stiffness in the cold may increase cardiac afterload contributing to the ventricular hypertrophy experienced by trout exposed to chronic low temperature [21,22,25,57].

### 4.1. Temperature associated changes in the compliance of the outflow tract

For the fish OFT to function as a windkessel, it must be highly compliant with specialized inflation properties [5]. At high stroke volume, the chamber expands to accommodate the increased volume of blood while maintaining a relatively constant outflow pressure. Equally, at low stroke volumes the bulbus must maintain an appropriate outflow pressure for blood to flow through the gill capillary network [5]. For the bulbus arteriosus to behave similarly at high and low cardiac output, it is most compliant near peak systolic pressure allowing the pressure change to plateau [5,11,18,25]. Thus, thermal remodelling of the fish bulbus arteriosus would be anticipated to effectively limit the impact of temperature-induced changes in cardiac stroke volume and pulsatile flow on ventral aortic blood flow. In the current study, the plateau phase of the r-shape filling curve began at approximately 0.25 ml volume and approximately 6 kPa pressure which is in agreement with peak systolic pressures recorded by Clark & Rodnick [25], for fish with similar mass and ventricular mass. The steep increase in pressure-to-volume occurred above approximately 6 kPa and approximately 0.4 ml filling volume. Following cold acclimation, the profile of the filling curve shifted slightly as at a 0.25 ml filling volume, pressure continued to rise more linearly with volume, giving a higher pressure to volume relationship through this phase of filling. Following warm acclimation, the plateau phase began at a lower filling volume (approx. 0.17 ml) and a lower pressure (approx. 4 kPa), compared to controls. This results in a lower pressure to volume ratio during this phase of filling.

Here, the pressure–volume relationship in the rainbow trout OFT showed increased chamber stiffness following cold acclimation and increased compliance following warm acclimation. An implication of our findings may be recruitment of the stiff collagen fibres in the adventitia at a lower volume [5]. These fibres resist further inflation of the bulbus but cause steep increases in intra-luminal pressure. Indeed, in our study, at the highest volumes within the physiological pressure range, intra-luminal pressure appeared to rise steeply, and this appeared to occur at a lower volume following cold acclimation. However, caution must be applied to interpretation of pressure–volume curves generated *ex vivo,* especially static curves as produced here*.* Braun *et al*. [5] suggest that the position of the bulbus arteriosus in the pericardium makes the third pressure phase of the pressure–volume curve unlikely to occur *in vivo* as the semi-rigid fish pericardium limits the extent to which the bulbus can expand [5,18]. It is also possible, however, that this restriction actually causes the final rise in pressure [5]. In addition, if the bulbus were to swell extensively it may interfere with atrial function, which would reduce cardiac output and, therefore, bulbus filling [5]. Indeed, the OFT preparations in this study only experienced the high-pressure phase at pressures approaching 10 kPa, which is the upper limit of physiological pressure ranges [38,39]. Further work is needed to assess the impact of thermal acclimation on dynamic pressure–volume curves from trout OFT where viscoelastic properties can be properly determined [5]. Interestingly, Clark & Rodnick [25] found no difference in bulbus compliance or distensibility with ventricular hypertrophy over a range of physiological pressures, suggesting that changes in cardiac pressure are alone not sufficient to trigger remodelling.

### 4.2. Structural remodelling of the fish OFT following temperature acclimation

The exceptional compliance and low tissue modulus of the bulbus arteriosus is due to the structure and organization of the chamber's connective tissue. The elastic fibres that make up approximately 70% of the chamber are in a loose fibrillar arrangement of low hydrophobicity, high solubility elastin [2,5,10,13,15,17,18]. This arrangement is distinct from mammalian arteries, where elastin is more amorphous and found as elastin-associated glycoprotein microfibrils [5,58]. In addition, the fish elastin is chemically and genetically unique [15,59,60]. A greater density of polar amino acids gives a low hydrophobic index which is likely to alter elastic recoil and reduce Young's modulus (a measure of the resistance to deformation under load) [5]. Here, we saw a decreased collagen-to-elastin ratio following warm acclimation which contributed to an increase in overall chamber compliance. At warm temperatures, the trout heart operates at low or routine stroke volumes [61]. Therefore, it is likely that the important part of the pressure–volume curve is at the low volumes, where high compliance is needed to maintain a physiological pressure range for blood flow. Additionally, acute warming increases fish heart rate *in vivo* as a direct (i.e. Q_10_ effect) on pace-making [62]. It is possible a more compliant bulbus aids rate of inflation to accommodate high heart rates at warm temperatures.

To maximize bulbar compliance there is minimal collagen in the media or luminal layers [10]. However, collagen integrates into the outer media, and the outer adventitia is almost entirely collagen as clearly seen in figure 2 (and see [2,5,8,63]). Fibrillar collagen monomers form fibrils which function as supra-molecular assemblies and in-turn form fibres [33]. Collagen provides strength to resist radial and/or longitudinal haemodynamic strain on the bulbar wall [5,14,64,65]. With cold acclimation, we found a higher collagen content which increased stiffness of the whole chamber. The increased collagen deposition may be a cardio-protective measure to prevent haemodynamic damage to the bulbus wall under the increased strain of receiving a high stroke volume of viscous blood. With high stroke volume, it is likely that the bulbus is operating in the high-volume range of the pressure–volume curve and thus must be strong enough to withstand high levels of inflation.

We found subtle shifts in amide I band FTIR spectra between warm- and cold-acclimated bulbus arteriosus tissue indicating a change in the biochemical profile in regions corresponding to collagen [44–46]. However, these shifts were minor suggesting that changes in collagen ordering are not the predominant mechanism for altering stiffness with thermal acclimation in this tissue. This conclusion is supported by the lack of significant changes in our index of collagen coherency. Rather, we found shifts related to changes in collagen fibre density [41–43] that could be underpinned by changes in MMP activity. Following warm acclimation, we found an increase in MMP-2 activity, which was probably due an increased MMP-2 to proMMP-2 ratio. As MMPs degrade collagen, increased MMP activity is associated with low tissue collagen content. Accordingly, following cold acclimation we found a decrease in MMP activity and a decrease in the proMMP-2 to MMP-2 ratio, which is associated with low levels of collagen degradation. Our gelatin zymography also identified an 83 kDa gelatinase which has previously been shown to have a similar activity to MMP-9, which directly digests gelatin [35,66]. These thermally induced changes in the regulators of fish collagens in the OFT are like that found previously in the trout ventricle and atria [21,24] indicating remodelling may occur concurrently across chambers in the trout heart. However, thermal acclimation has been shown to have differing effects in the ventricle of the zebrafish with cold suppressing collagen degradation genes [51]. These authors did not investigate corresponding changes in the zebrafish OFT.

### 4.3. Implications of changes in compliance of the fish OFT on the ventricle

The vertebrate heart is sensitive to load, with chronic changes in cardiac pressure or volume causing changes in size, form and function [67]. The ectothermic nature of fish means that temperature directly alters cardiac function and blood viscosity [19,23]. To counteract this, the heart and cardiovascular system of many fish remodel seasonally with changing ambient temperature [56]. As rainbow trout remain active throughout the winter, this remodelling response has generally been viewed as an adaptive response to the increased volume load of pumping cold viscous blood. However, recent studies have suggested aspects of the remodelling response are more consistent with a pressure overload, which in mammals, is generally considered maladaptive due to fibrosis [21,23]. Indeed, stiffening of the mammalian aorta is often associated with increased cardiac afterload and can cause ventricular hypertrophy and fibrosis [68]. Under normal circumstances, the fish bulbus arteriosus reduces afterload [14,39,63], however, reduced compliance and/or distensibility relative to ventricular output may cause increased cardiac afterload [25]. Indeed, reduced bulbar compliance in trout by parasite infection or cholecystokinin increases cardiac afterload leading to ventricular hypertrophy and fibrosis [39,69] and Clark & Rodnick [25] showed that increased cardiac afterload in fish induced ventricular hypertrophy via an increase in myocyte cross-sectional area, mirroring mammalian remodelling [25,57,70]. Hypertrophy (by increased myocyte diameter) and fibrosis are a consequence of cold acclimation of the muscular chambers of the fish heart [21,23,71], and are consistent with remodelling due, at least in part, to pressure overload [25,57,70,72].

## 5. Conclusion

We have previously shown that cold acclimation increases the stiffness of the contractile chambers of the trout heart, which is reduced following warm acclimation [21,24]. However, it was unclear if cold acclimation would affect the highly specialized and compliant OFT, which is made almost entirely of connective tissues. Here, we show when cold-acclimated tissue is compared with warm-acclimated tissue there was an increase in stiffness and an increase in the collagen-to-elastin ratio of connective tissue within the bulbus arteriosus. These findings add to our previous work in atria and ventricular tissue, suggesting a global increase in myocardial stiffness following cold acclimation and a global increase in myocardial compliance following warm acclimation. Finally, we suggest that increased stiffness of the OFT following cold acclimation contributes to the well documented cold-induced ventricular hypertrophy experienced by some fish. It thus appears that cold-dependent hypertrophic remodelling of the fish ventricle provides both cardio-protection from increased cardiac preload and afterload as well as compensation for reduced compliance of the OFT and increased cardiac afterload.

## Data accessibility

The data underpinning this paper has been deposited in Dryad: https://doi.org/10.5061/dryad.bzkh1898k.
